# Epidemiology and surgical management of breast cancer in gynecological department of Douala General Hospital

**Published:** 2012-10-19

**Authors:** Charlotte Tchente Nguefack, Martin Essomba Biwole, Annie Massom, Jacques Tsingaing Kamgaing, Theophile Nana Njamen, Gregory Halle Ekane, Thomas Egbe Obinchemti, Eugene Belley Priso

**Affiliations:** 1 Obstetrics and Gynecology Department, Douala General Hospital, Cameroon; 2 Radiation and Oncology Department, Douala General Hospital, Cameroon; 3 Surgery Department, Douala General Hospital, Cameroon

**Keywords:** Breast, cancer, epidemiology, surgery, Douala

## Abstract

**Introduction:**

Breast cancer is one of the most common gynecological cancers in our environment. Douala General Hospital (DGH) is one of the two main centers in Cameroon, where the cancerous patient can receive multidisciplinary management including radiotherapy.

**Methods:**

The aim of our study was to describe the epidemiological, clinical profile and surgical management of patients with breast cancer in gynecological department of DGH.

**Results:**

A total of 42 patients were recruited in our department within a period of 3 years (from November 2006 to October 2009). The mean age was 46 years (range: 29-73 years). Characteristics of our study group were as followed: female sex (100%); breast feeding (95.245%); familial history of breast cancer (7.14%); 14.29% of patients were nulliparous and 19.05% primiparous. The main mode of discovering the disease was auto examination (92.86%). The clinical tumor size ranges from 2cm to 20cm with a mean of 6.83cm. Patients were then mostly diagnosed at stage III (54.76%) of the WHO classification. Only 2.38% were diagnosed at stage I. The main method of diagnosis was breast fine needle aspiration. Neo adjuvant treatment was administered in 78.57% of patients and the main surgical treatment was mastectomy (92.86%). Many patients are still followed up (59.52%), but we already had a mortality rate of 14.29% at the end of December 2010. We had no feedback for 26.19% of the study group.

**Conclusion:**

Breast cancer is generally diagnosed in advanced stage in our milieu; there is therefore a need for generalized sensitization of the population.

## Introduction

The incidence of breast cancer has been progressively increasing worldwide. In France, 49 814 new cases were diagnosed in 2005 and it is the most frequent cancer in women (89 cases per 100 000 women) [1]. In USA, in 2007, 202 964 women were diagnosed with breast cancer [2]. In Cameroon, Mbakop and coll in 1997 found that breast cancer and cancer of the cervix, with the prevalence of 21.5% each, were the first two cancers of Cameroonian woman [3].

In developed countries, cancers are frequently diagnosed at an early stage allowing conservative treatment and reducing mortality due to a cancer. In countries with limited resources, locally advanced breast cancer is still common and has poor prognosis.

Douala General Hospital (DGH) is with the Yaoundé General Hospital, the two centers in Cameroon (Central Africa) equipped for complete management of breast cancer patients. We report the breast cancer cases operated in our department for the first three years we have been working in a multidisciplinary team in this hospital. The aim of our study was to describe the epidemiological, clinical profile and surgical management of patients with breast cancer.

## Methods

It was a descriptive study. We prospectively recruited 42 women consulting in our unit within a period of 3 years (November 2006 to October 2009). All patients consulting during this period for breast cancer were included except those lacking a pathological diagnosis.

We recorded socio demographic characteristics of patients (age, marital status, gravidity and parity, profession), their personal and family history. On clinical examination, the location of the lump was precised, it was measured and characterized. Other systems were examined in order to exclude secondary location of the disease. They were then sent to the laboratory for diagnostic tests (ultrasonography, mammography, fine needle aspiration or microbiopsy) and pre treatment work up (systematically, we ordered abdominal ultrasound, chest X-ray, full blood count, Ca 15-3, liver enzymes, bones X-ray for those who couldn't travel to Yaoundé for bone scintigraphy). After the work up, a staff meeting was organized between gynaecologist surgeon, oncologist and radiotherapist for the management options of each patient (according to the staging of their disease). Due to financial constraint, some poor patients could not do all the tests before treatment, in those cases, we insisted on pathology, chest X-ray, abdominal ultrasound and others according to clinical examination. The management consisted either of surgery, chemotherapy, radiotherapy and hormonotherapy or both according to the staging of their disease. The surgery was either radical or conservative (tumorectomy or quadrantectomy) with or without axillary nodes dissection. Statistics analyses were done using Le Sphinx plus2 software.

## Results

**Figure 1:**
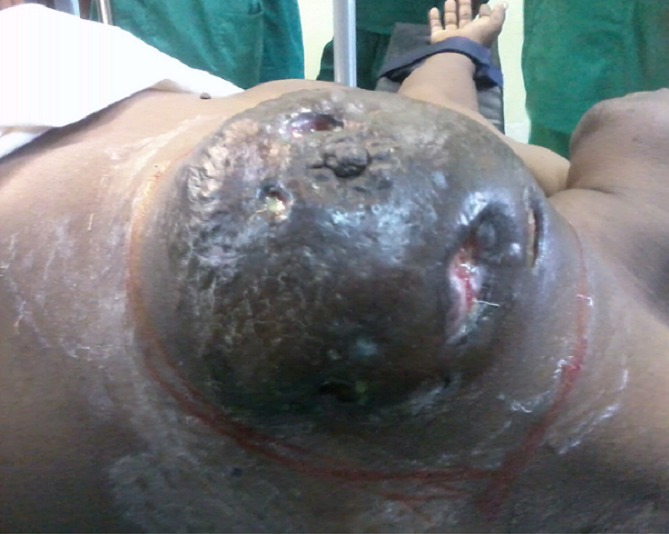
Patient with ulcerated breast cancer

**Figure 2:**
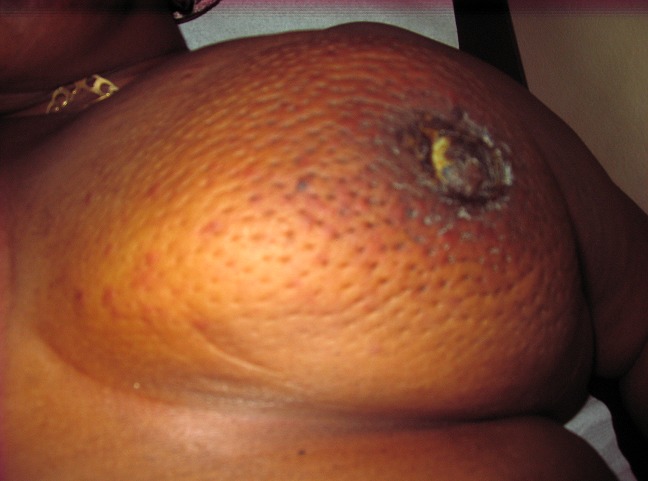
Patient with inflammatory breast cancer

**Figure 3:**
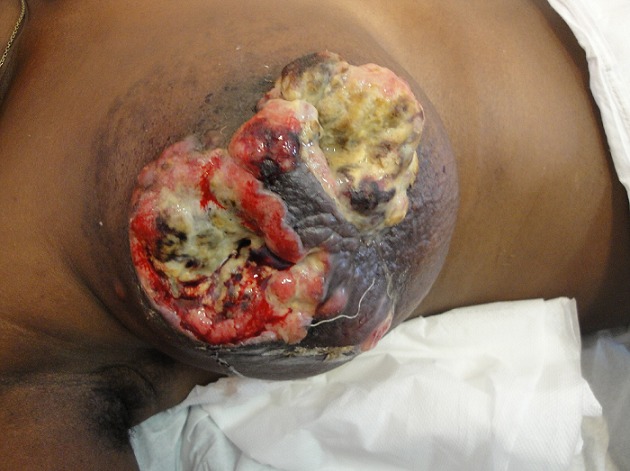
Young patient with ulcerated breast cancer

**Figure 4:**
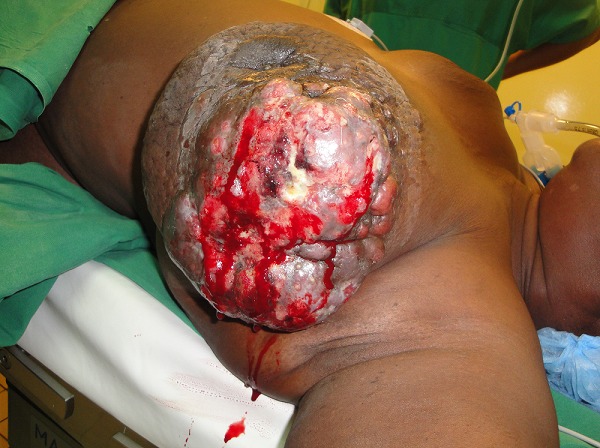
Patient with ulcerated breast cancer in the external quadran

**Figure 5:**
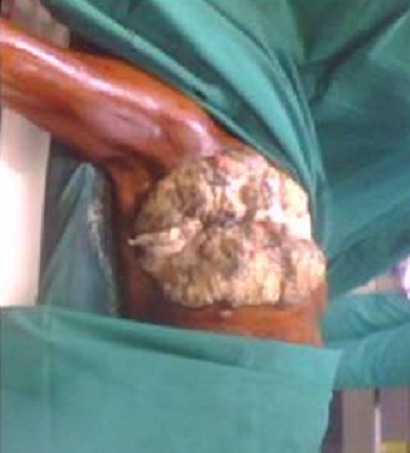
Proliferative tumour after first surgery

**Figure 6:**
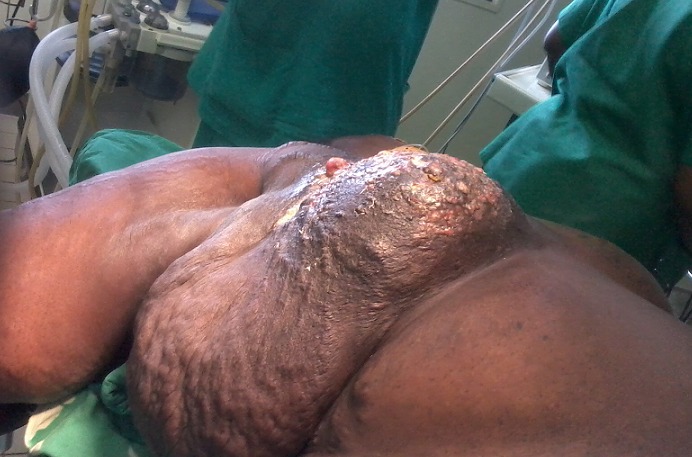
Ulcerative tumour

**Figure 7:**
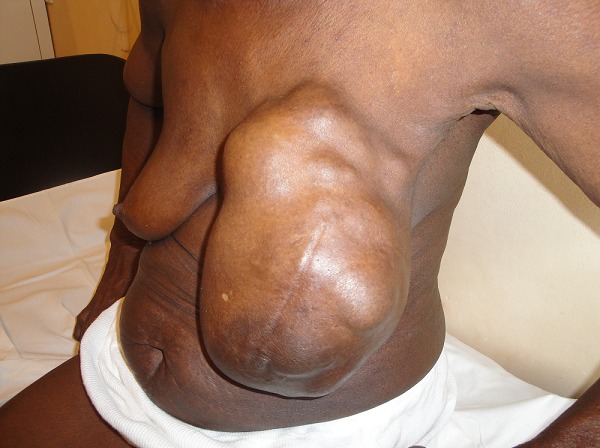
Tumour evolving more than one quadran of the breast

A total of 42 patients were recruited in our department within a period of 3 years from November 2006 to October 2009. The mean age was 46 years, range: 29 - 73 years. 36% of our patients were 30 to 40 years old and 60% were between 30 to 50 years (Table 1). Only 14.30% of patients were nulliparous; 35.71% were grand-multiparus (5 children or more) and 59.51% had 3 children or more. Concerning some risk factors presented by our patients, almost all women breastfed their children (95.24%). Three had a family history of breast cancer (7.14%); 11.90 had a past history of breast abscess; no patient had hormone replacement therapy. The majority of patients discovered their mass themselves (92.86%); 2.38% were diagnosed by a Doctor and 4.76% by a relative. No tumor was diagnosed by routine echography and mammography. The left breast was mostly involved (54.76%) as compared to the right (45.24%). The external Quadrant was the main location of the breast cancer: 57.14%, followed by the internal quadrant 21.83% (Table 2). The clinical tumor size ranges from 2cm to 20cm with a mean of 6.83cm.Tumors were mainly diagnosed at stage III (54.76%) of the WHO classification. Only 2.38% were diagnosed at stage I (Table 3, Figure 1, Figure 2, Figure 3, Figure 4, Figure 5, Figure 6, Figure 7). Majority of patients had fine needle aspiration as diagnostic technique (61.90%), the rest were diagnosed by micro biopsy (19.05%) or biopsy (19.05%). The common histological type of tumor was invasive ductal carcinoma (71.43%) (Table 4).

**Table 1:** Age distribution of a group of 42 women followed-up for breast cancer in the Gynecological Department of Douala General Hospital over a 3 year-period

Age group (Years)	numbers	%
< 30	1	2
30-40	15	36
41-50	10	24
51-60	12	29
61-70	3	7
>70	1	2
**Total**	**42**	**100**

**Table 2:** Breast location of the tumor in a group of 42 women followed-up for breast cancer in the Gynecological Department of Douala General Hospital over a 3 year-period

Location		Numbers	%
UEQ		15	37.71
LEQ		2	4.76
Union of EQ		7	16.67
	**Subtotal (EQ)**	24	57.14
UIQ		5	11.90
LIQ		2	4.76
Union of IQ		3	7.14
	**Subtotal (IQ)**	10	23.81
Retroareola		8	19.05
**Total**		42	100

UEQ: Upper External Quadrant, LEQ: Lower External Quadrant, UIQ Upper Inner Quadrant, LIQ: Lower Inner Quadrant

**Table 3:** Staging of the disease in a group of 42 women followed-up for breast cancer in the Gynecological Department of Douala General Hospital over a 3 year-period

Staging		Numbers	%
Stage I (1)		1	2.38
Stage II [12]	IIA	3	7.14
	IIB	9	21.43
Stage III (23)	IIIA	10	23.81
	IIIB	13	30.95
Stage IV (6)		6	14.29
**Total**		**42**	**100**

**Table 4:** Anatomopathology findings in a group of 42 women followed-up for breast cancer in the Gynecological Department of Douala General Hospital over a 3 year-period

		Numbers	%
**Mode of dg before surgery**	Fine needle aspiration	26	61.90
	Tru-cut (microbiopsy)	8	19.05
	biopsy	8	19.05
**Histological type**	IDC	30	71.43
	ILC	5	11.90
	Mucinous C	1	2.38
	Carcinoid Tumor	1	2.38
	Apocrine C	2	4.76
	adenocarcinoma	3	7.14

IDC : Invasive Ductal Carcinoma ; ILC : Invasive Lobular Carcinoma ; C: Carcinoma

Neoadjuvant chemotherapy was frequently administered (78.57%). Conservative surgery was rare (7.14%) comparing to mastectomy (92. 86%). Almost all patients received adjuvant therapy (Table 5). Many patients are still followed up (59.52%), but we already had a mortality rate of 14.29% at the end of December 2010. We had no feedback for 26.19% of the study group.

**Table 5:** Treatment in a group of 42 women followed-up for breast cancer in the Gynecological Department of Douala General Hospital over a 3 year-period

Treatment		Numbers	%
Neo adjuvant	Hormonotherapy	1	2.38
	Chemotherapy	32	78.57
Surgery	Simple mastectomy (in advanced ulcerated tumors)	5	11.90
	Mastectomy and axillary nodes dissection	34	80.95
	Quadrantectomy and axillary nodes removal	3	7.14
Adjuvant therapy	Chemotherapy	39	92.86
	Radiotherapy	41	97.62
	Hormonotherapy	39	92.86

## Discussion

From November 2006 to October 2009, 42 patients were operated for breast cancer in our unit. The mean age was 46 years; 36% of them were 30 to 40 years old and 60% were between 30 and 50years. This could be explained by the fact that the Cameroonian population is predominantly young. The average age is 22.1 years and life expectancy was 50.364 years in 2010 [4]. It should however be noted that Brinton LA and al in the USA have observed a higher incidence of breast cancer in the young black compared to the young white for the age group under 40 years [5]. Young age has been established as a factor of poor prognosis of breast cancer in numerous studies [6–8]. According to Shannon and al in their review on breast cancer in young women, young age was independently prognostic factor when tumor size, nodal status, grade, hormone receptor status, loco regional treatment and adjuvant systemic therapy were taken into account [7].

Our patients were essentially multiparous (52.38% of women had at least 3 children) and 95.24% of women breastfed. The low parity, conventionally regarded as a risk factor for breast cancer is not found here, along with the benefits of breastfeeding [9]. Doh and al observed similar findings with respect to multiparity and breastfeeding [10]. In USA, Hall and al realized that multiparity was associated with increased risk of breast cancer among younger African-American women but not among younger white women [11]. This is based on the theory that a transient increase in risk follows pregnancy (Within 3 years), and to subsequent long-term protective effect post-pregnancy for 10 years; generally because of the differentiation of breast duct. Since African-American women have more children at young ages and at intervals of 3 years or less, the transient risk period could be prolonged.

Three patients out of 42 (7.14%) had a family history of breast cancer. The research for a BRCA1 and BRCA2 mutation gene was not done because there is no genetic lab available in our country. It is very cost effective to have it done abroad.

Clinically, the majority of our patients had a consultation done at an advanced stage of the disease: WHO stage III (54.86%). Ekortarl A and Ndom in their study of 20 women with advanced cancer in Cameroon found that, the primary reasons given for waiting so long to seek care were: inability to pay for medical care (10 patients); misdiagnosis by General Practitioners leading to time loss before coming for consultation (9 patients); beliefs, fears, cultural factors, ignorance (9 patients). More than one factor could be identified in half of the patients [13]. All these reasons were found in this study.

Concerning the pathology, much remains to be done in our laboratories which are not well equipped to study the characteristics of the tumors (No immunohistochemistry and other techniques of molecular and genetic analysis). Therefore, our results only described the histological type: 71.43% where infiltrating ductal carcinomas. As a result of the advanced stage at diagnosis, adjuvant chemotherapy was almost systematically administered (92.86%) as well as radiotherapy and hormone therapy (97.62% and 92.86% respectively). Ovarian ablation or suppression with or without tamoxifen is an effective endocrine therapy in the adjuvant treatment of breast cancer in women with estrogen receptor (ER) positive or ER-unknown breast cancer [14]. No ovarian ablation was done in our study.

Surgery was radical in 92.85% of cases because of late diagnosis. With the sensitization of women and the organisation of mass screening, cancers could be diagnosed at early stages and allow conservative treatments. None of our patients was diagnosed through screening. For a majority (92.86%), it was breast auto examination. Meanwhile, in the Western countries, screening organisation has reduced mortality due to breast cancer [15]. After 3 years, 14.29% of patients died in our study group and 26.29% were out of sight (alive or dead?). The prognosis was then poor, probably due to the advanced stage of the disease at diagnosis.

## Conclusion

About 15 years after the first publications on breast cancer in our country: we still have a majority of cases diagnosed at late stages leading to radical surgery. It is obvious that there is need for health education, training and organization of campaigns for breast cancer screening; we still have difficulties in getting good pathology result. Many prognostic factors are lacking: hormone receptors, Her2 neu, Ki67, etc.; we really need to think about all this and improve, especially because of the younger age of women with breast cancer in our environment; screening may start at 40 years instead of 50 in our milieu.

## References

[1] Enjeux médicaux des cancers http://www.inserm.fr/thematiques/cancer/enjeux/enjeux-medicaux.

[2] US. Cancer Statistics Working Group (2010). United States Cancer Statistics: 1999–2007 Incidence and Mortality Web-based Report. http://www.cdc.gov/uscs.

[3] Mbakop A, Yomi J, Mouele SA (1997). Les cancers au Cameroun. Guide pratique. CEPER.

[4] http://www.statistics-cameroon.org/downloads.

[5] Brinton LA, Sherman ME, Carreon JD, Anderson WF (2008). Recent trends in breast cancer among younger women in the United States. J Natl Cancer Inst..

[6] van der Sangen MJ, Voogd AC (2008). Breast cancer in young women: epidemiology and treatment dilemmas. Ned Tijdschr Geneeskd..

[7] Shannon C, Smith IE (2003). Breast cancer in adolescents and young women. European Journal of Cancer..

[8] Fowble BL, Schultz DJ, Overmoyer B (1994). The influence of young age on outcome in early stage breast cancer. Int J Radiat Oncol Biol Phys..

[9] Helewa M, Lévesque P, Provencher D (2002). Breast cancer, pregnancy, and breastfeeding. J Obstet Gynaecol Can.

[10] Doh AS The women and her breast over the years.

[11] Hall IJ, Moorman PG, Millikan RC, Newman B (2005). Comparative Analysis of Breast Cancer Risk Factors among African-American Women and White Women. Am J Epidemiol..

[12] Haffty BG, Silber A (2006). Racial differences in the incidence of BRCA1 and BRCA2 mutations in a cohort of early onset breast cancer patients: African American compared to white women. J Med Genet..

[13] Ekortarl A, Ndom P, Sacks A (2007). A study of patients who appear with far advanced cancer at Yaoundé General Hospital, Cameroon, Africa. Psychooncology..

[14] Carlson RW, Anderson BO (2003). Treatment of breast cancer in countries with limited resources. Breast J.

[15] Hoerger TJ, Ekwueme DU (2011). Estimated effects of the national breast and cervical cancer early detection program on breast cancer mortality. Am J Prev Med..

